# Physiological roles of chloride ions in bodily and cellular functions

**DOI:** 10.1186/s12576-023-00889-x

**Published:** 2023-11-15

**Authors:** Yoshinori Marunaka

**Affiliations:** 1 Medical Research Institute, Kyoto Industrial Health Association, General Incorporated Foundation, 67 Kitatsuboi-Cho, Nishinokyo, Nakagyo-Ku, Kyoto, 604-8472 Japan; 2 Research Organization of Science and Technology, Ritsumeikan University, Kusatsu, 525-8577 Japan; 3 Graduate School of Medical Science, Kyoto Prefectural University of Medicine, Kamigyo-Ku, Kyoto, 602-8566 Japan

**Keywords:** Cl^−^, Cell proliferation, Cell cycle, Neurite elongation, GTPase, WNK

## Abstract

Physiological roles of Cl^−^, a major anion in the body, are not well known compared with those of cations. This review article introduces: (1) roles of Cl^−^ in bodily and cellular functions; (2) the range of cytosolic Cl^−^ concentration ([Cl^−^]_c_); (3) whether [Cl^−^]_c_ could change with cell volume change under an isosmotic condition; (4) whether [Cl^−^]_c_ could change under conditions where multiple Cl^−^ transporters and channels contribute to Cl^−^ influx and efflux in an isosmotic state; (5) whether the change in [Cl^−^]_c_ could be large enough to act as signals; (6) effects of Cl^−^ on cytoskeletal tubulin polymerization through inhibition of GTPase activity and tubulin polymerization-dependent biological activity; (7) roles of cytosolic Cl^−^ in cell proliferation; (8) Cl^−^-regulatory mechanisms of ciliary motility; (9) roles of Cl^−^ in sweet/umami taste receptors; (10) Cl^−^-regulatory mechanisms of with-no-lysine kinase (WNK); (11) roles of Cl^−^ in regulation of epithelial Na^+^ transport; (12) relationship between roles of Cl^−^ and H^+^ in body functions.

## Introduction

The ionic environment in the body plays an important role in bodily and cellular function [1–10]. The physiological role of cations has been studied in detail; *i.e.*, Na^+^, K^+^, Ca^2+^, Mg^2+^ and H^+^ are well known to contribute to cellular functions such as generation of action potential, maintenance of resting membrane potential and control of enzyme activity. On the one hand, the most well-recognized role of Cl^−^, the major anion in the body, is as a counter ion to the cation for maintenance of electroneutrality, but other physiological significance of Cl^−^ is currently only marginally recognized.

Active Cl^−^ transport such as Na^+^-K^+^-2Cl^−^ transporter (NKCC) and K^+^-Cl^−^ transporter (KCC) is involved in the maintenance of the membrane potential [1–5]. NKCC contributes to water secretion into the luminal side of epithelial tissues driven by elevating luminal osmolarity via active transcellular Cl^−^ secretion in concert with Cl^−^ channels followed by the paracellular Na^+^ secretion [11, 12]. One of the most famous diseases based on impaired water secretion due to dysfunction of Cl^−^ channels is cystic fibrosis (CF) in the lung, the liver, sinus, small and large intestines, pancreatic and hepatobiliary ducts, and male reproductive tracts [13–18]. CF is a genetic disease caused by mutations in cystic fibrosis transmembrane conductance regulator (CFTR), the cloning of which was performed in 1989 [19]. The main cause of death in CF patients is pulmonary infection due to drying of the lung cavity as a result from insufficient water secretion caused by dysfunction of certain Cl^−^ channels. A Cl^−^ channel was cloned as cystic fibrosis transmembrane conductance regulator (CFTR) [19], and CFTR has been characterized by many researchers [20]. Furthermore, the recently proposed role of CFTR as a modulator of immune tolerance may explain the presence of persistent portal vein inflammation leading to fibrosis, and the enterohepatic axis would also be involved in the presentation and progression of the disease [18]. In addition, Cl^−^ also plays physiological roles in regulation of activities of enzymes, gene expression, ion channels, ion transporters, ion pumps, ion environments, infection prevention, etc.; *e.g.*, GTPase activity [21], neurite elongation [22], resistance against anticancer drugs [23–25], cell death [26, 27], regulation of cell volume [28], autophagy [29, 30], cell proliferation [31], ciliary movements [32–34], sweet/umami taste sensing at sweet/umami taste receptors [35, 36], WNK activity [37], epithelial Na^+^ transport [38, 39], mRNA expression of epithelial Na^+^ channel (ENaC) [40–43], the Na^+^,K^+^-pump activity [44], and pH homeostasis cooperating with bicarbonate [45–47].

This review will introduce physiological roles of Cl^−^, molecular mechanisms of Cl^−^ actions on a variety of physiological phenomena, and possibilities that cytosolic Cl^−^ may act as intracellular signals.

## Requirements for cytosolic Cl^−^ to function as intracellular signals

For cytosolic Cl^−^ to function as intracellular signals, Cl^−^ must cause conformational changes in proteins, such as enzymes, which regulate intracellular functions. For this to happen, the cytosolic Cl^−^ concentration ([Cl^−^]_c_) must change or the binding affinity of Cl^−^ to these proteins must change. Since only a limited number of studies have been conducted so far to precisely investigate how the binding affinity of Cl^−^ to various proteins is altered by any factors, this review article will primarily address whether [Cl^−^]_c_ can in fact be altered and, if so, to what extent [Cl^−^]_c_ must be altered for cytosolic Cl^−^ to act as intracellular signals.

## How much is [Cl^−^]_c_?

The [Cl^−^]_c_ in gastric and respiratory epithelial cells is reported to be about 50 mM [48–50]. Several studies have also reported that [Cl^−^]_c_ in the paranasal olfactory system (plough nose) cells are very variable, 5 ~ 80 mM [51–60]. [Cl^−^]_c_ is known to be higher in the dendrites of neurons than in the cell bodies [51, 61]. Further, Engels et al. [60] has reported that under both control and chemical ischemia conditions, [Cl^−^]_c_ values markedly differ in various subcellular regions and cell types. Their study [60] also indicates that the [Cl^−^]_c_ in astrocytes of the hippocampal cornu ammonis region 1 is 21 mM, which is lower than that (28 mM) in dentate gyrus, but higher than that (14 mM) in neocortical astrocytes. In addition, the [Cl^−^]_c_ in radial glia-like cells (20 mM) is comparable to the value (21 mM) of astrocytes in the hippocampal cornu ammonis region 1 [60]. These [Cl^−^]_c_ values (14 ~ 28 mM) [60] are considerably much lower than 35 mM [Cl^−^]_c_ determined in cerebellar Bergmann glia cells [59]. The studies [48, 49, 51–61] indicate that [Cl^−^]_c_ values are very valuable depending on types and regions of cells. One of the most important points is whether [Cl^−^]_c_ changes under an isosmotic condition; *i.e.*, it should be considered whether [Cl^−^]_c_ changes under an isosmotic condition due to the following reason. As Cl^−^ moves, cations such as Na^+^ and K^+^ also move in the same direction as Cl^−^ to maintain electrical neutrality, causing osmolarity elevation. This osmolarity elevation causes water movement in the same direction. Therefore, the increase in [Cl^−^]_c_ due to Cl^−^ movement is attenuated by the water movement. Thus, the change in [Cl^−^]_c_ due to Cl^−^ movement under an isosmotic condition should be carefully evaluated.

## Does [Cl^−^]_c_ change with cell volume change under an isosmotic condition?

**Fig. 1:**
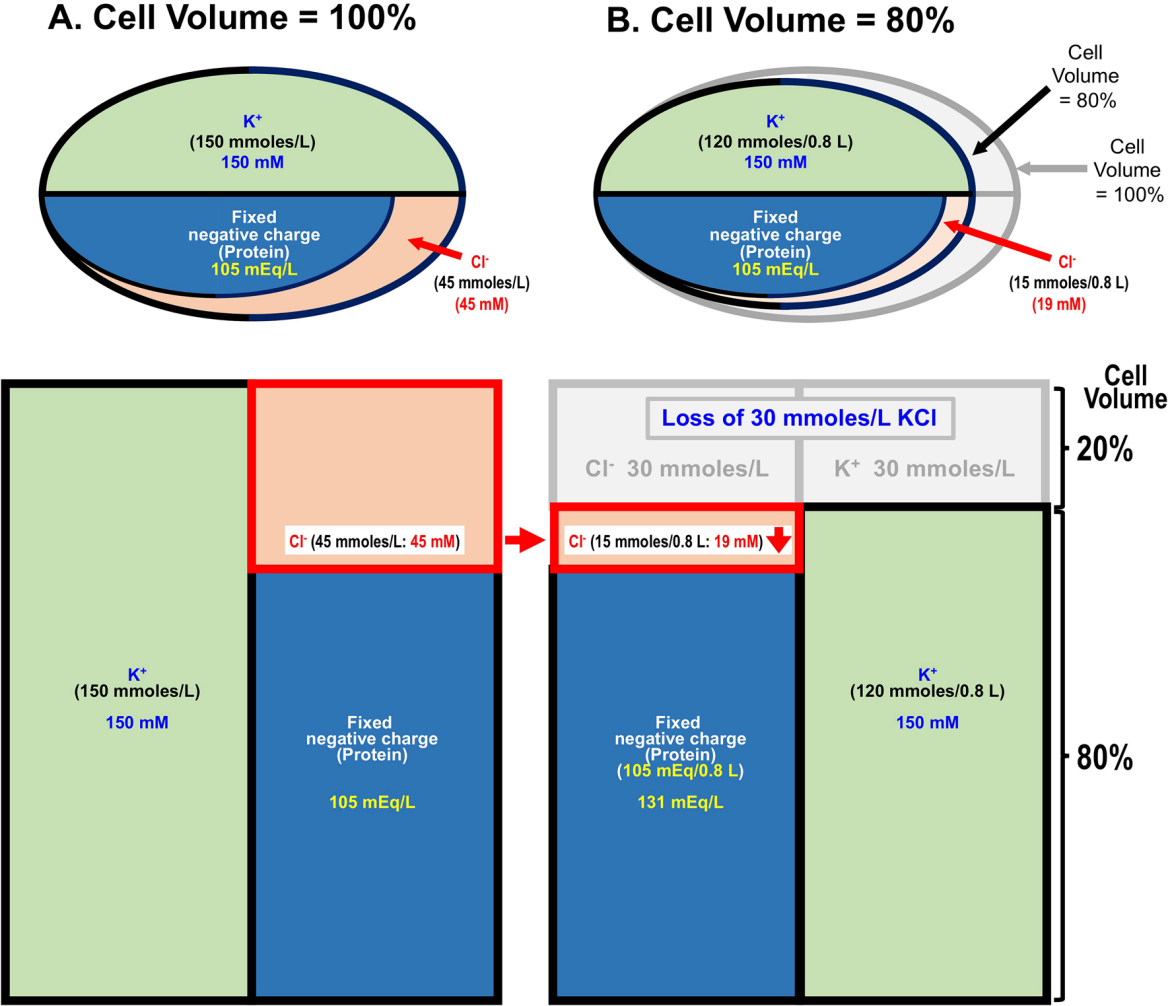
A simple model of correlation between changes in cytosolic Cl^−^ concentration ([Cl^−^]_c_) and cell volume under isosmotic conditions. This simple model shows contents and concentrations of K^+^, Cl^−^ and membrane-impermeable fixed negative charges such as proteins, although other ions including Na^+^ and HCO_3_^−^ significantly exist in cell. **A** Cell volume = 100%. Cell contains 150 mmoles/L K^+^ (150 mM), 45 mmoles/L Cl^−^ and 105 mEq/L fixed negative charges. **B** Cell volume = 80%. When the cell volume reduces to 80% under an isosmotic condition, 20% K^+^ (30 mmoles/L) is released from the cytosolic space to the extracellular space; K^+^ remaining in the cytosolic space is 120 mmoles/L and the cytosolic K^+^ concentration (150 mM) is same as before the cell volume change occurs. On the one hand, when cell volume decreases by 20%, the same amount of Cl^−^ (30 mmoles/L) as K^+^ must be released from the cytosolic space to the extracellular space to keep electroneutrality. This means that the cytosolic Cl^−^ is reduced to 15 mmoles/L from 45 mmoles/L after the occurrence of 20% cell volume decrease, and the cytosolic Cl^−^ concentration is reduced to 19 mM (15 mmoles/0.8 L) from 45 mM before the cell volume decrease occurs. This is because even though the cytoplasmic Cl^−^ content (45 mmoles/L) is much lower than the cytoplasmic K^+^ content (150 mmoles/L), the same amount of Cl^−^ as K^+^ must be released from the cytosolic space due to the presence of a large amount of membrane-impermeable fixed negative charge

Changes in [Cl^−^]_c_ are discussed based on the relationship between water movement and cell volume associated with Cl^−^ movement. One of the requirements for cytosolic Cl^−^ to act as an intracellular signal is a change in [Cl^−^]_c_. It is recognized that unlike cytosolic Ca^2+^ concentration ([Ca^2+^]_c_), [Cl^−^]_c_ may not change much. Furthermore, it is easy to imagine that changes in the extracellular fluid osmolarity would induce water movement in and out of the cell, which would change [Cl^−^]_c_. On the other hand, can the [Cl^−^]_c_ change under an isosmotic condition of extracellular fluids? Cytosolic K^+^ concentration ([K^+^]_c_) generally does not change regardless of the amount of K^+^ efflux (or influx) in a state in the isosmotic state. This is due to the efflux (influx) of anions (generally Cl^−^) with an equivalent negative charge to maintain cytosolic electroneutrality at cationic K^+^ movement, followed by water movement to compensate for osmotic changes caused by these ion (K^+^ and Cl^−^) movements (Fig. 1). As a result, no change in [K^+^]_c_ occurs. On the other hand, [Cl^−^]_c_ changes under these circumstances unlike [K^+^]_c_ (Fig. 1). In the case of anions, [Cl^−^]_c_ is kept lower than [K^+^]_c_ due to the presence of many anions (various proteins) in the cell that cannot pass through the plasma membrane (membrane-impermeable anions such as big molecule proteins: so-called fixed charges) (Fig. 1). The presence of many membrane-impermeable anions (proteins) causes [Cl^−^]_c_ to change in response to K^+^ efflux (or influx) [1] (Fig. 1). This is because even if [Cl^−^]_c_ is lower than [K^+^]_c_, Cl^−^ efflux (or influx) occurs in the same amount as the K^+^ efflux (or influx) in order to maintain cytosolic electroneutrality, since Cl^−^ is the major membrane-permeable anion in the cytosolic space under the condition that large amounts of membrane-impermeable anions (fixed charges) are present (Fig. 1).

## Does [Cl^−^]_c_ changes under the condition that multiple Cl^−^ transporters and channels contribute to Cl^−^ influx and efflux in an isosmotic state?

Cl^−^ transport across the plasma membrane by Cl^−^ transporters and channels participates in changes of [Cl^−^]_c_. Cl^−^ transport can be broadly divided into active and passive transport systems. The most well-known active Cl^−^ transporters are Na^+^-K^+^-2Cl^−^ cotransporter (NKCC), Na^+^-Cl^−^ transporter (NCC), Na^+^-driven Cl^−^/HCO_3_^−^ exchanger (NDCBE) and K^+^-Cl^−^ cotransporter (KCC) [62–65]. NKCC and NCC contribute to Cl^−^ uptake into cells using electrochemical potential of Na^+^ generated by the Na^+^,K^+^-pump (ATPase), while NDCBE and KCC, respectively, participate in Cl^−^ extrusion from cells using electrochemical potential of Na^+^ and K^+^ generated by the Na^+^,K^+^-pump (ATPase).

An increase in NKCC-mediated Cl^−^ influx elevates [Cl^−^]_c_. The elevated [Cl^−^]_c_ induces an increase in the chemical potential of cytosolic Cl^−^, which elevates Cl^−^ efflux through Cl^−^ channels. The [Cl^−^]_c_ increases until the elevation of Cl^−^ efflux through Cl^−^ channels is equal to the increase in Cl^−^ influx via NKCC. At this point, the [Cl^−^]_c_ change reaches equilibrium. The opposite phenomenon occurs when NKCC-mediated Cl^−^ influx is reduced; [Cl^−^]_c_ decreases, causing reduction of Cl^−^ efflux through Cl^−^ channels. Then, the reduction in [Cl^−^]_c_ reaches equilibrium at a point when the decrease in Cl^−^ efflux through Cl^−^ channels is equal to the reduction of Cl^−^ influx via NKCC. Further, when activities of KCC change, similar phenomena occur, resulting in alternation of [Cl^−^]_c_. In addition, altering Cl^−^ channel activity causes the same thing in [Cl^−^]_c_. An increase in Cl^−^ channel activity induces elevation of Cl^−^ flux (influx or efflux depending on the electrochemical potential of cytosolic Cl^−^), which changes [Cl^−^]_c_. This change in [Cl^−^]_c_ alters the chemical potential of cytosolic Cl^−^, which changes the Cl^−^ flux through Cl^−^ channels and settles at the same point as the original Cl^−^ flux through Cl^−^ channels. Thus, even though multiple Cl^−^ transporters and channels contribute to Cl^−^ influx and efflux, [Cl^−^]_c_ changes when activities of Cl^−^ transporters and/or channels are altered. An interesting point in terms of Cl^−^ flux is that changes in [Cl^−^]_c_ due to changes in Cl^−^ channel activity are transient, whereas changes in Cl^−^ flux due to changes in Cl^−^ transporter activity are persistent.

## Is the change in [Cl^−^]_c_ large enough to act as an intracellular signal?

Consideration should be given whether the change in [Cl^−^]_c_ is large enough to act as an intracellular signal. [Ca^2+^]_c_ increases about tenfold compared to the resting state, while [Cl^−^]_c_ usually changes only about twofold (or 0.5-fold) [66]. Is it possible that Cl^−^, which fluctuates only over such a small range of change, could play an intracellular signaling role? The binding number of Cl^−^ to target substances such as channel proteins and enzymes should be also considered. If the number of Cl^−^ binding sites on a protein with enzyme activity is 3, a twofold change in Cl^−^ concentration ([Cl^−^]) has the same effect on enzyme activity as an eightfold change (2^3^ = 8: 2, twofold change in [Cl^−^]; 3, the number of binding sites). When the number of Cl^−^ binding sites is 4, a twofold change in [Cl^−^] has the same effect as a 16-fold change in [Cl^−^] (2^4^ = 16: 2, twofold change in [Cl^−^]; 4, the number of binding sites). The theoretical simulation suggests that a small [Cl^−^]_c_ change such as twofold would be large enough to act as an intracellular signal.

## Effects of Cl^−^ on cytoskeletal tubulin polymerization through inhibition of GTPase activity and tubulin polymerization-dependent biological phenomena

Because [Cl^−^]_c_ in GABAergic neurons is generally lower than that estimated when the membrane potential is set to the equilibrium potential of Cl^−^, the GABA-induced increase in the conductance of Cl^−^ channel causes Cl^−^ influx, resulting in membrane hyperpolarization, which develops during maturation [67–69]. The lower [Cl^−^]_c_ in GABAergic neurons is maintained by KCC. However, in some cases of GABAergic neurons, GABA causes membrane depolarization by inducing an Cl^−^ efflux via an increase in the Cl^−^ channel conductance. The GABA-induced Cl^−^ efflux is attributed to an actual [Cl^−^]_c_ higher than that predicted when the membrane potential is taken as the equilibrium potential for Cl^−^ and this higher [Cl^−^]_c_ is maintained by NKCC. For example, in 'juvenile' neurons, GABA induces Cl^−^ efflux from the cytosolic space to the extracellular one, and causes membrane depolarization [62, 69–73]. Over the course of development, the GABA-induced change in Cl^−^ flux shows transition from efflux to influx [71]: GABA induces Cl^−^ efflux due to a high [Cl^−^]_c_ causing membrane depolarization in immature stages, while GABA induces Cl^−^ influx due to a low [Cl^−^]_c_ leading to membrane hyperpolarization in mature stages [71, 74]. The maturation-induced decrease in [Cl^−^]_c_ is due to a change in functional expression of Cl^−^ transporters contributing to Cl^−^ uptake such as NKCC to KCC participating in Cl^−^ extrusion. Here, significance of high [Cl^−^]_c_ in immature stages should be considered [67–69, 75–83]. Of course, excitatory signals of GABAergic stimulation cause depolarization of the plasma membrane and an increase in [Ca^2+^]_c_ via activation of voltage-gated Ca^2+^ channels. In addition, high [Cl^−^]_c_ in immature stages would be required for the formation of neural networks via tubulin polymerization and its stability [84–87] as described below.

**Fig. 2:**
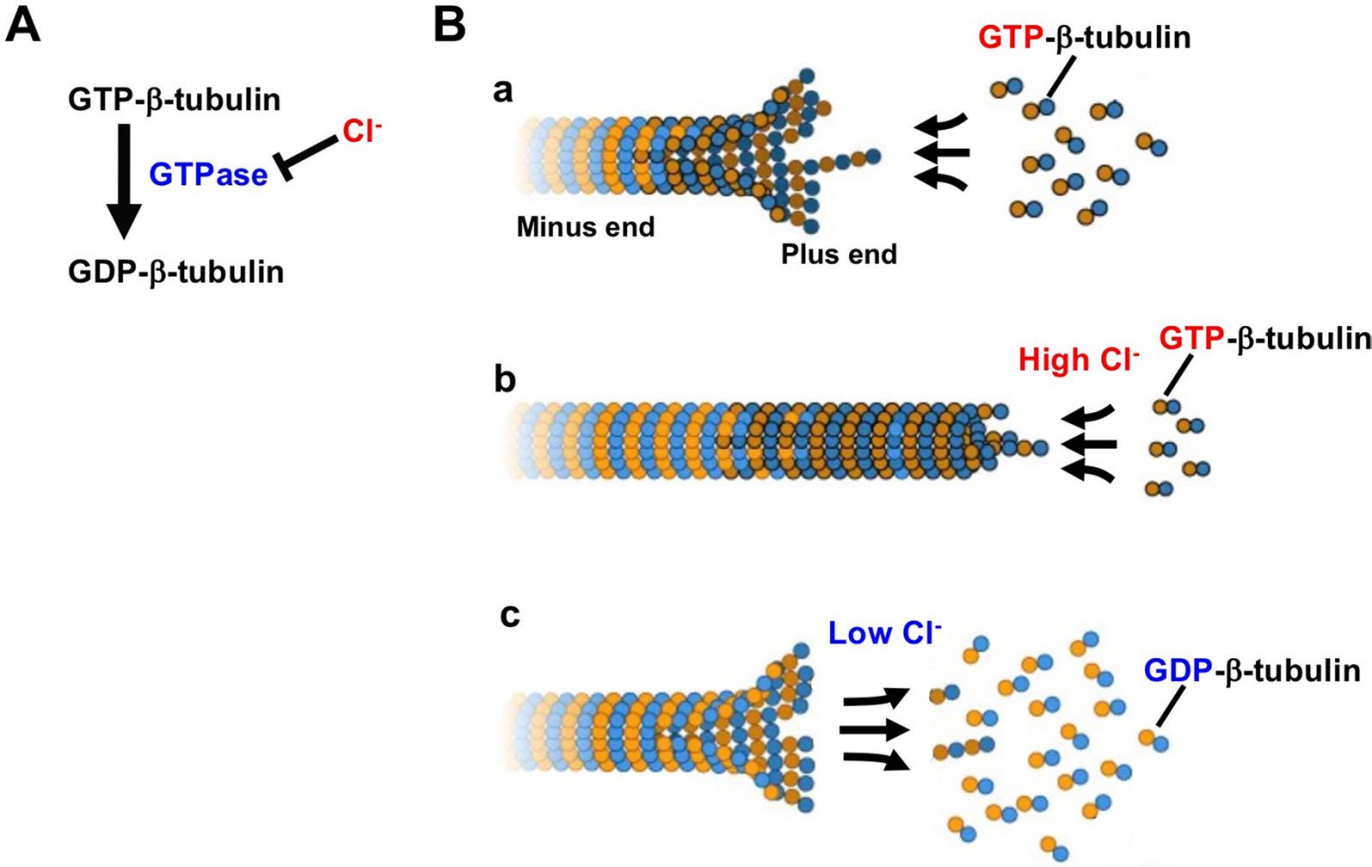
Action of Cl^−^ on dynamics of tubulin polymerization and depolymerization. **A** Cl^−^ suppresses conversion of GTP-ß-tubulin to GDP-ß-tubulin by inhibiting GTPase. **B** a In most cells, tubulin is present in concentrations of 10–20 μM, favoring the assembly of microtubules at the plus end. In filaments with slower growth rates, there is less lateral interaction between protofilaments. The inherent curvature of the GDP-tubulin dimer surface gives the appearance of fraying at this end. b High [Cl^−^]_c_ stabilizes GTP-ß-tubulin by blocking GTPase activity [21], and leads to a condition of a higher concentrations of GTP-tubulin dimers. Thus, tubulin polymerization is promoted at the plus end by forming a rigid GTP-cap. c GTPase increases at low [Cl^−^]_c_. This leads to concerted GTP hydrolysis, weakening the tubulin dimer interactions, and rapidly disassembling tubulin polymerization. This figure is produced using the result obtained in a report by Nakajima et al. [21] combining information shown in ‘What is microtubule dynamic instability?’ by MBINFO DEFINING MECHANOBIOLOGY (see the information shown in “https://www.mechanobio.info/cytoskeleton-dynamics/what-is-the-cytoskeleton/what-are-microtubules/what-microtubule-dynamic-instability/#what-is-microtubule-dynamic-instability”) under a Creative Commons Attribution-NonCommercial 4.0 International License

Cl^−^ has the ability to attenuate GTPase activity [21, 48, 66, 88–90] (Fig. 2A). Inhibition of GTPase promotes polymerization of tubulin, a type of cytoskeleton [21, 91–93] (Fig. 2A). Tubulin monomers are subclassified into three categories: *i.e.*, α, ß, and γ subtypes [85, 94–97]. Polymerization of tubulin is formed by the binding of α- and ß-tubulin subtypes: the α/ß-tubulin heterodimer has two GTP-binding sites; one located on ß-tubulin (the E site) and the other on α-tubulin (the N site) [85, 98–102]. ß-tubulin has GTPase activity that hydrolyzes GTP during polymerization, and then produces GDP [85–87, 103] (Fig. 2A). This GDP is still bound to ß-tubulin, which is part of the tubulin polymer [85–87]. The GDP bound to ß-tubulin at depolymerization is exchanged to GTP, and GTP-bound ß-tubulin can polymerize once more [85–87, 103] (Fig. 2B). In contrast, the GTP bound to the N site in α-tubulin is neither hydrolyzed to GDP nor exchangeable to GDP during tubulin polymerizing/depolymerizing dynamics [85, 98, 103] (Fig. 2A and B). Moreover, the amino acid residues, Asp 251 and Glu 254, in α-tubulin stimulate the GTPase activity of ß-tubulin [84, 85, 87, 104]. The microtubule dynamic is achieved when the GTP molecule in the E site of ß-tubulin is hydrolyzed [85, 86]: Growth and stability in microtubule are facilitated by the presence of a “GTP cap” at its ( +), which is required in order for α and ß tubulin to stably bind to each other and promote polymerization [84, 85] (Fig. 2B-a). Like β-tubulin, actin subunits also have intrinsic GTPase activity related to microfilament stability [84]. Cl^−^ has the ability diminishing GTPase activity [21, 48, 66, 88–90] (Fig. 2A). Therefore, Cl^−^ at high concentrations inhibits the intrinsic GTPase activity of tubulin, thus preventing GTP degradation and promoting tubulin polymerization by stably binding GTP to tubulin [66, 88] (Fig. 2B-b). This suggests that cytosolic Cl^−^ plays key roles in cellular functions by modulating tubulin-polymerization states such as formation of neuronal connectivity and network in immature stages [76, 81, 87] cancer aggressiveness, cell death, cell migration, invasion, and sensitivity to chemotherapy [107], meiosis [108], triggering of dynamic improvement in cell plasticity, regulation of energy transfer [86], and cardiac mechanics [109]. Thus, Cl^−^-induced promotion of the tubulin polymerization [21] serves as a plasma membrane lining structure in neurite outgrowth, and promotes plasma membrane elongation, which is essential for neurite outgrowth, resulting in neurite outgrowth [21, 110]. In fact, it has been reported that neurites lengthen as [Cl^−^] increases [110]. Activation of Cl^−^ uptake into the cytosolic space in neurons enhances elongation of neurite [112, 113], while elevation of Cl^−^ release from the cytosolic space in neurons negatively regulates elongation of neurite [113].

In the early stage of cell division, microtubules composed of tubulin elongate from the centrosome (microtubule forming center), leading to cell division [115, 116]. The process of tubulin depolymerization (Fig. 2B-c) is then required for the ‘correct’ cell division at the end of cell division [115, 116]. Taxanes exert their anticancer effects by blocking tubulin depolymerization, thereby preventing cell division and arresting cancer cell division and proliferation [107, 117–120]. Cytosolic Cl^−^ plays an important role in the action of taxanes [48]. When [Cl^−^]_c_ decreases, endogenous GTPase activity in ß-tubulin increases, leading to enhancement of the degradation of GTP to GDP and the depolymerization of tubulin (Fig. 2B-c). Therefore, at low [Cl^−^]_c_, taxanes cannot inhibit tubulin depolymerization, thus taxanes cannot show their action as anticancer drugs [48], although low [Cl^−^]_c_ slows the cell proliferation rate [31, 121–124].

Further, it has been reported that Cl^−^ channels participate in resistance against anticancer drugs [23–25]. For instance, impaired activity of volume-sensitive Cl^−^ channel (volume-sensitive, outwardly rectifying (VSOR) Cl^−^ channel or volume-regulated anion channel (VRAC)) is involved in resistant potentials against an anticancer agent, cisplatin [23, 25]. Further, it has been reported that an increase in expression of ClC-3 Cl^−^ channel would activate the NF-κB, leading to expression of P-glycoprotein, a type of ATP-binding cassette [24, 25]. P-glycoprotein plays an important role in the formation of so-called multidrug resistance by extruding anticancer drugs such as paclitaxel belonging to the taxanes [24, 25]. Thus, the ClC-3 Cl^−^ channel would play an important role in the mechanism generating multidrug resistance [24, 25]. Another study [124] has also elucidated that ClC-3 Cl^−^ channels generate paclitaxel resistance in ovarian cancer. In paclitaxel-resistant ovarian cancer cells (A2780/PTX cells), ClC-3 Cl^−^ channels are found to be upregulated in both their protein expression and function compared with their parental A2780 cells [124]. The diminution of ClC-3 Cl^−^ channel expression by siRNA in A2780/PTX cells has partly recovered the sensitivity to paclitaxel by causing the G_2_/M arrest via diminution of ClC-3 Cl^−^ channel function and elevation of tubulin polymerization [124]. Paclitaxel also shows its blocking action on the current through ClC-3 Cl^−^ channels in A2780 cells, but not in A2780/PTX cells [124]. These observations suggest us that the Cl^−^ current (flux) through ClC-3 Cl^−^ channel influences paclitaxel potential on tubulin polymerization by regulating [Cl^−^]_c_ which participates in the sensitivity to paclitaxel. Furthermore, paclitaxel is known to cause damage of plasma membrane, leading to cell death in ovarian A2780 cancer cells [125]. Of interest, paclitaxel also induces cell swelling in ovarian A2780 cancer cells, causing pyroptosis [126, 127], which is one of cell death types, apoptosis, pyroptosis, and necrosis [128, 129]. Pyroptosis has been first reported in macrophages infected with Salmonella typhimurium [126]. The molecular mechanism producing pyroptosis is investigated from a biochemical viewpoint regarding gasdermins, a family of pore-forming proteins in humans [130, 131]. Cytosolic Cl^−^ should be also considered to be a key player in pyroptosis via control of WNK (see Sect. "Cl^-^-regulatory mechanisms of with-no-lysine kinase (WNK) and its physiological role") [132, 133]. Induction of apoptosis by activation of Cl^−^ channels has been demonstrated by Shimizu et al. [133] (also refer to [27, 135]). Lee et al. [23] have reported that downregulation of VRAC is involved in multidrug resistance (also refer to [27, 135]). Further, VRAC contributes to lipopolysaccharide plus nigericin-induced pyroptosis in bone marrow-derived macrophages [135–137]. Thus, cytosolic Cl^−^ and various types of Cl^−^ channels including ClC-3 Cl^−^ channel and VRAC are suggested to be key players involved in cell death and drug resistance.

## Roles of cytosolic Cl^−^ in cell proliferation

In gastric cancer cells, G_0_/G_1_ arrest is induced by decreased [Cl^−^]_c_ [31, 121–124, 139–152]. G_0_/G_1_ arrest induced by decreased [Cl^−^]_c_ is regulated via retinoblastoma protein (Rb) by a p53-independent, p21-dependent mechanism [120]: lowering [Cl^−^]_c_ upregulates expression of p21, resulting in a decrease in CDK2 expression, which diminishes Rb phosphorylation, thus G_0_/G_1_ arrest occurs [120] (see a review article [152] regarding cell proliferation via p53-p21 dependent regulation).

Chloride intracellular channel 1 (CLIC1) is also reported to be a key factor in cell proliferation of esophageal squamous cell carcinoma [153–155]. Further, a cohort study [156] reports that genetic polymorphism in methylenetetrahydrofolate reductase Cl^−^ transport protein 6 (MTHFR CLCN6) gene is associated with keratinocyte skin cancer, suggesting a role of Cl^−^ in proliferation of human skin cancer. Thus, Cl^−^ is one of key factors controlling cell proliferation.

## Cl^−^-regulatory mechanisms of ciliary motility

**Fig. 3:**
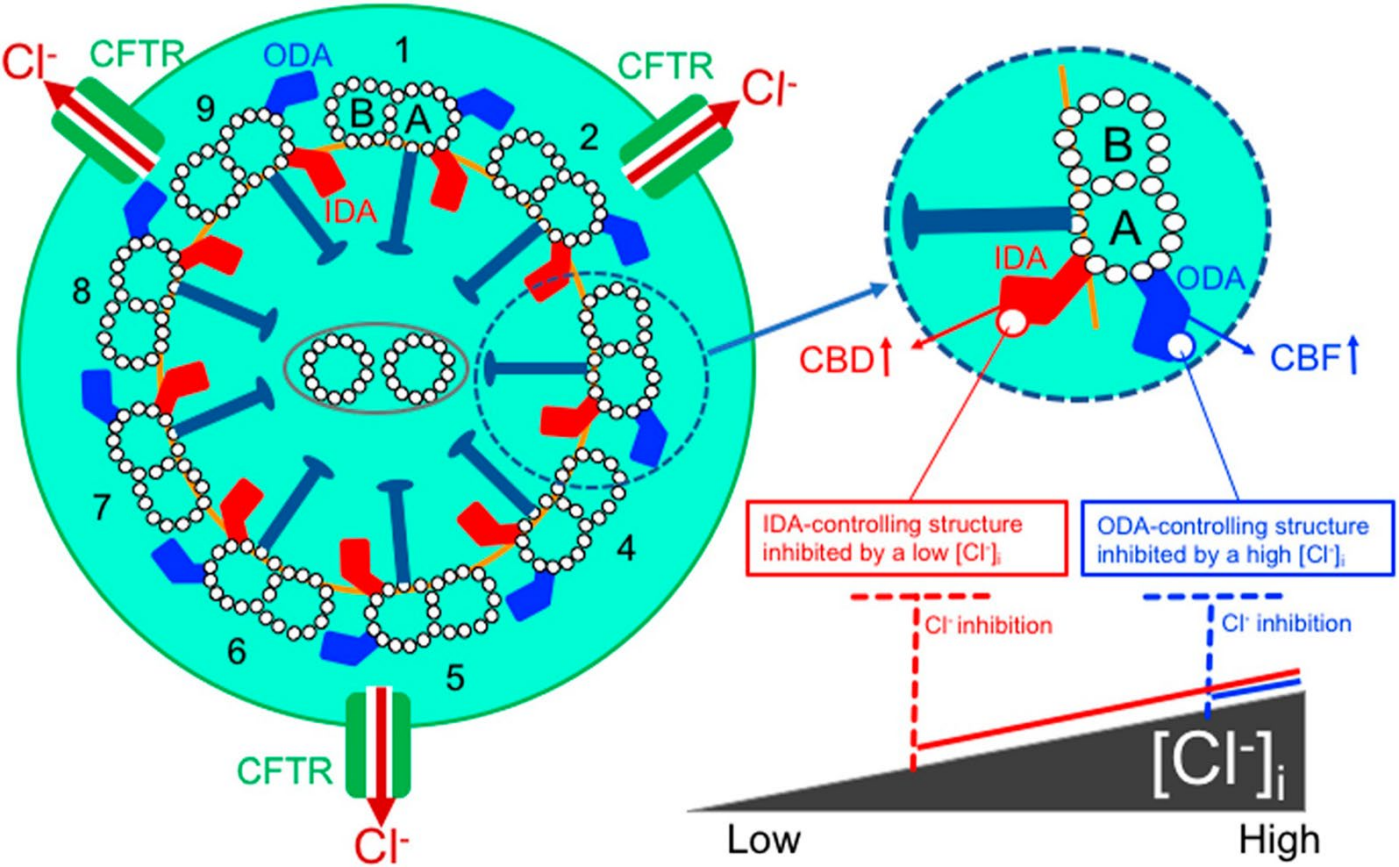
Cl^−^-regulation of ciliary beating in cultured human nasal epithelial cells. Outer dynein arms (ODAs) control the frequency of ciliary movement, and inner dynein arms (IDAs) control waveform including the amplitude (angle) of ciliary movement. Cytosolic Cl^−^ inhibits, respectively, the function of ODAs and IDAs via binding to Cl^−^ biding sites in the axonemal structures of ODAs and IDAs reducing the frequency and the amplitude (angle) of ciliary movement. The sensitivity of ODAs to cytosolic Cl^−^ is less than that of IDA. Cytosolic Cl^−^ at basal levels has no inhibitory effect on ODA function, but inhibits IDA function to some extent, but not completely. When [Cl^−^]_c_ becomes lower than the basal level, ODA, which is not inhibited by basal levels of Cl^−^, maintains its activity, and IDA, which is partially inhibited by basal levels of Cl^−^, is no longer inhibited by Cl^−^ and its activity is increased. Conversely, as [Cl^−^]_c_ increases, the activity of both ODA and IDA decreases. This figure is modified from Fig. 8 reported in a study [168] under a Creative Commons Attribution-NonCommercial 4.0 International License

Ciliary movement in the airways is essential for the function of the body's defense system by expelling foreign substances that enter the airways from the body through dynein-driven mechanisms [157] via cAMP-mediated pathways [158–163], protein kinase C-mediated pathways [164] and cytosolic Ca^2+^-mediated pathways [166, 167] in addition to water secretion from airway epithelia driven by Cl^−^ secretion [88]. Cilia also play an important role in cerebrospinal flow [167]. The activity of ciliary movement is evaluated by two indices: (1) the amplitude (angle) of ciliary movement and (2) the frequency of ciliary movement (the number of ciliary movements per unit time) [32, 34, 169–171]. The amplitude and frequency of the ciliary movement are, respectively, controlled by the inner dynein arm (IDA) and outer dynein arm (ODA) [33, 169, 172, 173] (Fig. 3). Cytosolic Cl^−^ has been reported to suppress both amplitude and frequency of ciliary movement, with the inhibitory action of Cl^−^ on amplitude reaching the maximum level at low [Cl^−^]_c_ and on frequency at high [Cl^−^]_c_ [32–34]. Beta-agonist-induced cell shrinkage activates ciliary movements via a decrease in [Cl^−^]_c_ (Fig. 1) [174]. Elevation of [Ca^2+^]_c_ also enhances ciliary movements [176, 177]. These phenomena suggest that the Cl^−^ sensors in IDA controlling the amplitude of ciliary movement are more sensitive to Cl^−^ than Cl^−^ sensors in ODA controlling the frequency of ciliary movement [168] (Fig. 3).

In sperm, mitochondrion forms a complex with rat sarcoma (Ras)-associated binding (Rab) protein 10 (RAB10) of active form (GTP bound form of RAB10; GTP-RAB10) via TOMM20 (a subunit protein of translocase in the outer membrane of mitochondria) [177–179]. GTP-RAB10 binds to dynein, which transports mitochondrion by forming a complex of DNAH7, a tubulin-related protein [179]. GTPase-activating protein (GAP) and guanine nucleotide exchange factor (GEF) are well known as the main factors regulating the activity of small GTP-binding proteins [181, 182]. GEF facilitates the dissociation of GDP-binding in small GTP-binding proteins, which is subsequently replaced by GTP [181, 182]. On the one hand, GAP activates the GTPase of small GTP-binding proteins, enhancing the conversion of GTP to GDP, leading to inactivation of small GTP-binding proteins [179–181]. ADP-ribosylation factor 6 (ARF6) is a GTPase [182]. GTP-locked mutant ARF6^QL^ of ARF6 binds to dynein, enhancing autophagic vesicles transport in neurons, while GDP-locked mutant ARF6T27N of ARF6 impairs retrograde transport of autophagic vesicles along the axon [183, 184]. Taken together, when the GTP-bound form of dynein-binding protein binds to dynein, it promotes the transport capacity of dynein.

Cl^−^ attenuates GTPase activity [21, 48, 66, 88–90], therefore high Cl^−^ stabilizes GTP-bound form of dynein-binding protein. On the one and, cytosolic Cl^−^ inhibits the amplitude of ciliary movement at low [Cl^−^]_c_, and the frequency of the ciliary movement at high [Cl^−^]_c_ [32, 169] (Fig. 3). These observations indicate that Cl^−^ of high concentration inhibits ciliary movements despite enhanced dynein transport activity by stabilizing active GTP-bound form of dynein-binding protein. From these observations, it is speculated that, unlike the unidirectional transport by dynein, the periodic appearance of GTP- and GDP-bound forms of dynein-binding proteins regulates ciliary reciprocal movement, although further direct evidence is needed to conclude the speculation.

## Roles of Cl^−^ in sweet/umami taste sensing at sweet/umami taste receptors

Taste is a chemical sensation perceived on the tongue and proved by chemicals in foods and beverages, and plays a very important role in the ingestion of foods and other substances, as well as in sustaining life [184–187]. Cl^−^ is considered to participate in taste sensing, however details of molecular mechanisms of Cl^−^ involvement are unknown [35]. Recently, a Cl^−^ binding site has been reported in taste receptor type 1 (T1r), a member of the sweet and umami receptors on the tongue [35]. The T1r2a/T1r3 heterodimer found in medaka fish, the only T1r currently available for structural analysis, has a Cl^−^ specific binding site near the amino acid binding site in the ligand binding domain (LBD) of T1r3, which is likely conserved among species including human T1r3. This Cl^−^ binding at sub-millimolar to low millimolar concentrations induces a conformational change in the ligand-binding domain of the T1r2a/T1r3LBD [35]. Further, a small amount of Cl^−^ bound to the sweet and umami taste receptors stimulates the neuron connected to the receptor cells, suggesting a small amount (low concentration) of Cl^−^ enhances sweet and umami taste [35]. This finding strongly supports what has been said so far that small amounts (low concentrations) of NaCl promote sweet sensitivity [36].

## Cl^−^-regulatory mechanisms of with-no-lysine kinase (WNK) and its physiological role

WNK is named for the fact that it, a serine/threonine kinase, lacks a catalytic lysine in subdomain II, which serves an important role as an ATP binding site [188]. WNK regulates the activity of stress-related serine-threonine kinases, STE20 (sterile 20)/SPS1-related proline/alanine-rich kinase (SPAK) and oxidative stress-responsive kinase 1 (OSR1), which are targets of WNK signaling, and consequently regulates cellular functions by modulating activities of PI3K-AKT, TGF-ß, and NF-κB [189]. WNK also regulates cation-coupled Cl^−^ cotransporters via SPAK/OSR1 activation in renal epithelia, such as NKCC (NKCC1 and NKCC2) and KCC (KCC1—KCC4) [65, 191–198], which play crucial roles in regulation of the body fluid contents and blood pressure. NKCC and KCC are also regulated by various factors including flavonoids like quercetin and myricetin [40, 66, 88, 111, 199–205], which also show various actions including anti-diabetic and anti-hypertensive ones [66, 203, 206]. Activity of WNK is controlled by Cl^−^ [206]. Low [Cl^−^]_c_ activates WNKs, which phosphorylate the paralogous Ste20 kinases, SPAK/OSR1, on a T-loop threonine (Thr 233 in SPAK, Thr 185 in OSR1) to activate the kinases, SPAK/OSR1 [207]. The activated SPAK/OSR1 phosphorylate NKCC1, NKCC2 and NCC on serine/threonine conserved in N-termini of the transporters [207]. Activated SPAK/OSR1 phosphorylate serine/threonine conserved at the N-terminus of NKCC1, NKCC2 and NCC, increasing the transporting activity of the transporters [207]. WNK has two domains, a smaller N-terminal domain and a larger C-terminal domain, which form an inactive, asymmetric dimer [207]. Cl^−^ binds to its binding site in the subunit of unphosphorylated dimeric WNK, stabilizing the inactive dimer of WNK [207], thus Cl^−^ inhibits WNK activity [207, 208]. The physiological meaning of Cl^−^-induced stabilization of inactive dimer of WNK is that a decrease in [Cl^−^]_c_ activates NKCC and NCC that participate in Cl^−^ uptake into the intracellular space via enhancement of WNK phosphorylation (activation), contributing to the homeostasis of [Cl^−^]_c_.

**Fig. 4:**
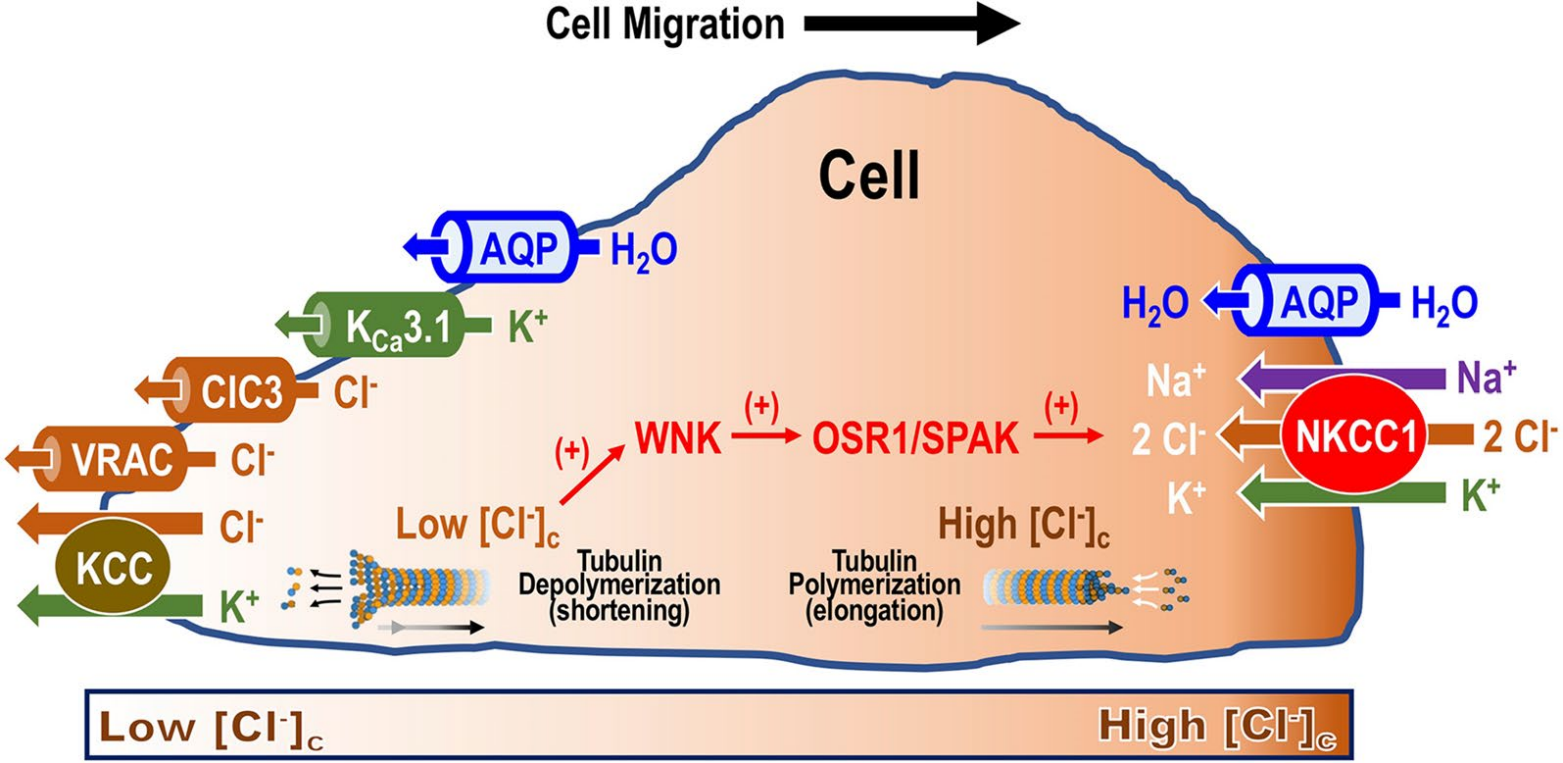
Roles of cytosolic Cl^−^, ion transporters and ion/water channels in cell migration. When cells migrate, cells need to change cell shape. Na^+^-K^+^-2Cl^−^ cotransporter 1 (NKCC1) and aquaporin (AQP) are expressed on the migrating side membrane. NKCC1 is involved in Cl^−^ uptake into the cytosolic space with Na^+^ and K+ [197, 209–212]. The uptake of these ions results in an influx of water into the cytosolic space via AQP through an increase in cytosolic osmolarity [196]. The movement of Cl^−^, Na^+^, K^+^ and water causes an increase in cell volume accompanied with [Cl^−^]_c_ elevation, which promotes tubulin polymerization (elongation) [212] by inhibiting GTPase activity (see Fig. 2B). Similar to tubulin polymerization, actin monomers are enhanced to be polymerized. Then, cells migrate via these processes. On the one hand, K^+^-Cl^−^ cotransporter (KCC), volume-regulated anion channel (VRAC), Ca^2+^-activated K^+^ channel (K^+^_Ca_3.1) and AQP are expressed on the tail end membrane of cell migration and excretes Cl^−^ with K^+^ to the extracellular space via KCC, VRAC and K^+^_Ca_3.1 [197, 212]. Water efflux to the extracellular space via AQP is caused by a decrease in cytosolic osmolarity due to excretion of these ions. The movement of Cl^−^, K^+^ and water results in a decrease in cell volume accompanied with [Cl^−^]_c_ diminution, which leads to tubulin depolymerization (shortening) at the tail end of the cell migration [212] by activating GTPase (see Fig. 2C). WNK activated by lowered [Cl^−^]_c_ induces phosphorylation (activation) of OSR1/SPACK, which increases activity of NKCC1 by phosphorylating NKCC1 [208]. Thus, WNK is importantly involved in cell migration [208]

When cells migrate, cells need to change cell shape. WNK activated by lowered [Cl^−^]_c_ induces phosphorylation (activation) of SPAK/OSR1, which increases activity of NKCC1 by phosphorylating NKCC1, then activated WNK participates in cell migration as follows [208]. NKCC1 is expressed on the front line side of cell migration and is involved in the uptake of Cl^−^ into the cytosolic space along with Na^+^ and K^+^ (Fig. 4) [197, 209–212]. The uptake of these ions results in the water influx via aquaporin (AQP) into the cytosolic space by increasing cytosolic osmolarity [196]; *i.e.*, the movement of Cl^−^, Na^+^, K^+^ and water results in cell expansion (an increase in cell volume) with elevation of [Cl^−^]_c_ (Fig. 1), which stimulates polymerization (elongation) of tubulin in high [Cl^−^]_c_ areas (Fig. 4) [212] by inhibiting GTPase activity (see Fig. 2A and B-b). Through these processes, cells migrate toward the front. On the one hand, KCC, volume-regulated anion channel (VRAC), Ca^2+^-activated K^+^ channel (K^+^_Ca_ 3.1) and AQP are expressed at the tail end of cell migration and excretes Cl^−^ along with K^+^ to the extracellular space [197, 212]. Water efflux to the extracellular space via AQP is caused by a decrease in cytosolic osmolarity due to excretion of these ions; the movement of Cl^−^, K^+^ and water results in a decrease in cell volume with diminution of [Cl^−^]_c_ (Fig. 1), which leads to depolymerization (shortening) of tubulin in low [Cl^−^]_c_ areas (Fig. 4) [212] by activating GTPase (see Fig. 2A and B-c).

## Roles of Cl^−^ in regulation of epithelial Na^+^ transport

Dinudom and Cook et al. [38, 39] have reported that as [Cl^−^]_c_ rises from 5 to 150 mM, the amplitude of the inward Na^+^ current declines via G protein G_i_ and G_o_ subclasses-mediated pathways by applying whole-cell patch-clamp techniques to the cells of the intralobular ducts of the mandibular glands of mice. Tohda et al. [49] have also reported that reduction in [Cl^−^]_c_ caused by activation of Ca^2+^-activated K^+^ channels and cAMP-activated Cl^−^ channels (see Fig. 1) increases the open probability (P_o_) of amiloride-sensitive Na^+^-permeable channels in lung epithelial cells treated with ß_2_ agonist by using patch clamp single channel recording and single cell imaging techniques. Further, the reduction in [Cl^−^]_c_ caused by ß_2_ agonist has been reported to play an essentially important role in activation of amiloride-sensitive Na^+^-permeable channels in lung epithelial cells treated with ß_2_ agonist [213], suggesting that the cytosolic Cl^−^ decreases the activity of amiloride-sensitive Na^+^-permeable channels in lung epithelial cells treated with ß_2_ agonist. A molecular model of the cytosolic Cl^−^-induced inhibition on amiloride-sensitive Na^+^ channels in ß_2_-agonist-treated lung epithelial cells has been proposed [214]. These reports [49, 214, 215] indicate that Cl^−^ has physiologically important roles in epithelial Na^+^ transport. On the one hand, using single channel recording techniques, Yamada et al. [215] have reported that lowering pipette (extracellular) Cl^−^ concentration decreases the P_o_ of single ENaC expressed on the apical membrane of renal cells treated with arginine vasotocin (AVT) to 0.23 ± 0.02 from 0.30 ± 0.02 associated with a significant decrease in the open time from 0.78 ± 0.03 to 0.61 ± 0.02 s without any significant change in the closed time. Further, the activity of the Na^+^-K^+^ pump has been reported to depend on the Cl^−^ conductance of the membrane on which the Na^+^-K^+^ pump is expressed [44], and this Cl^−^ conductance-dependent Na^+^-K^+^ pump activity is regulated via PTK activity [44].

Expression of ENaC mRNA is also regulated by cytosolic Cl^−^ [40–43]. Activation of Na^+^-K^+^-2Cl^−^ cotransporter (NKCC) by flavonoids such as apigenin and quercetin or diminution of Cl^−^ efflux by Cl^−^ channel blockers such as (5-Nitro-2-(3-phenylpropylamino)benzoic acid (NPPB) diminishes mRNA expression of ENaC in renal epithelial cells via elevation of [Cl^−^]_c_. [40–49]. Further, hypotonicity applied to ENaC-expressed renal cells elevates mRNA expression of ENaC in renal epithelial cells via diminution of [Cl^−^]_c_ by activating p38 MAPK and inducing MKP-1-mediated suppression of ERK [42, 43]. The hypotonicity-induced activation of p38 MAPK and suppression of ERK via MKP-1 would be mediated at least partially by the hypotonicity-induced decrease in [Cl^−^]_c_ [66, 88, 203].

## Relationship between roles of Cl^−^ and H^+^ in body functions

**Fig. 5:**
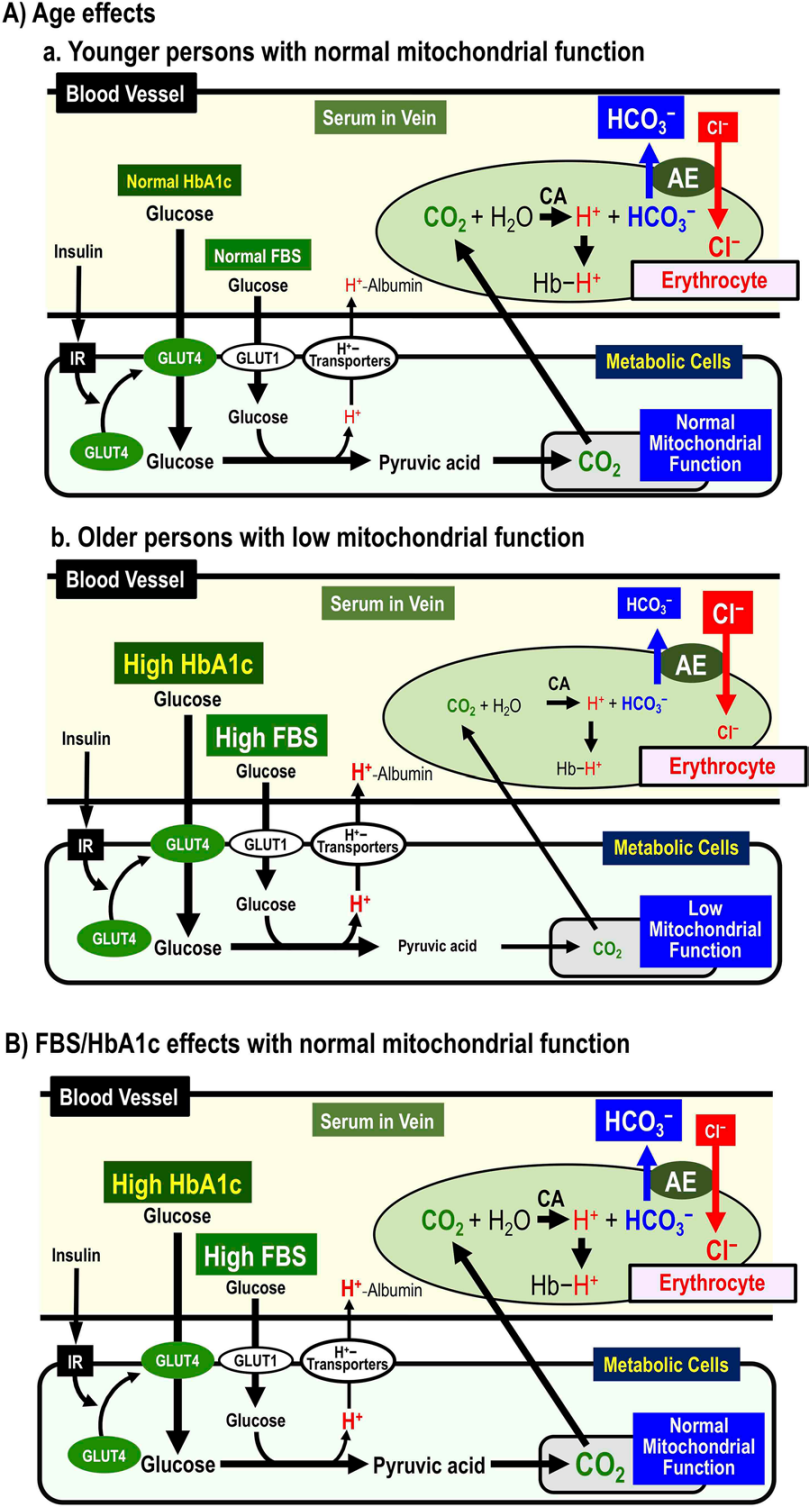
Aging- and FBS-dependent regulatory mechanisms of body fluid pH via transporting systems of Cl^−^ and HCO_3_^−^. **A** Age effects on venous serum Cl^−^ concentration ([Cl^−^]_s_). a Younger persons with normal mitochondrial function. Glucose is metabolized into pyruvic acid, and then CO_2_ is produced from the pyruvic acid in mitochondria with normal function. The produced CO_2_ moves into erythrocytes, and is converted into H^+^ and HCO_3_^−^ via a CA-facilitated process. The HCO_3_^−^ is exchanged with serum Cl^−^ via a Cl^−^/HCO_3_ anion exchanger (AE). These processes lead to low [Cl^−^]_s_. b Older persons with low mitochondrial function. The amount of CO_2_ produced in mitochondria becomes low due to low mitochondrial function. Thus, the amount of H^+^ and HCO_3_^−^ produced from CO_2_ becomes low. These processes keep high [Cl^−^]_s_. **B** FBS/HbA1c effects on [Cl^−^]_s_. with normal mitochondrial function. Glucose is metabolized into pyruvic acid, and then CO_2_ is produced from the pyruvic acid in mitochondria with normal function. The produced CO_2_ moves into erythrocytes, and is converted into H^+^ and HCO_3_^−^ via a CA-facilitated process. The HCO_3_^−^ is exchanged with serum Cl^−^ via a Cl^−^/HCO_3_ anion exchanger (AE). In cases of high FBS/HBA1c with normal mitochondrial function, large amounts of CO_2_ are produced, resulting in production of large amounts of HCO_3_^−^. These processes lead to low [Cl^−^]_s_. Figure 5 has been originally published in Marunaka et. al. (2021) Int J Mol Sci 22:11111 [237] under a Creative Commons Attribution-NonCommercial 4.0 International License

pH is lowered by H^+^ and CO_2_ produced in metabolic cells such as myocytes, neurons, etc., H^+^ is produced via glycolysis, and also from CO_2_ (CO_2_ ↔ H^+^  + HCO_3_^−^) via TCA cycle in mitochondria: the produced H^+^ plays various important roles in cellular functions [201, 217–237]. Lowered pH disturbs various body functions such as appearance of insulin resistance, enhancement of cancer metastasis and elevation of amyloid-ß production [201, 217–235]. In peripheral tissues, CO_2_ produced by metabolic cells moves to capillary erythrocytes, where CO_2_ is converted to H^+^ and HCO_3_^−^ via a carbonic anhydrase (CA)-facilitated process. H^+^ binds to Hb in erythrocytes, while HCO_3_^−^ is exchanged for blood extracellular Cl^−^ (outside erythrocytes) by anion exchanger (AE; Cl^−^/HCO_3_^−^ exchanger), and is excreted out of erythrocytes; this Cl^−^ movement is the so-called ‘Cl^−^ shift’ (Fig. 5). Thus, the extracellular Cl^−^ concentration in veins is lower than that in arteries. These phenomena mean that the serum Cl^−^ concentration in veins would be an indicator of metabolic status and mitochondrial function. Although the serum HCO_3_^−^ concentration is a more direct indicator than the serum Cl^−^ concentration, the process of accurately measuring the serum HCO_3_^−^ concentration is clinically cumbersome and it is not practical to easily measure the serum HCO_3_^−^ concentration in many humans. In fact, the venous serum Cl^−^ concentration has been reported to be an indicator of metabolic status and mitochondrial function in analysis of over 100,000 healthy humans [237]. It is interesting to note that in ‘healthy’ individuals, the venous Cl^−^ concentration increases with age, whereas the venous serum Cl^−^ concentration decreases with increasing fasting blood glucose (sugar: FBS) and HbA1c [237]. These phenomena suggest that in ‘healthy’ individuals aging diminishes mitochondrial function (lowering CO_2_ production, and leading to lower the venous serum HCO_3_^−^ concentration coupled with a higher venous serum Cl^−^ concentration [237] (Fig. 5A-a for younger persons and Fig. 5A-b for older persons); furthermore, ‘healthy’ individuals with high FBS and HbA1c levels may have higher intracellular glucose concentrations and consequently higher mitochondrial CO_2_ production, leading to elevate the venous serum HCO_3_^−^ concentration coupled with a lower venous serum Cl^−^ concentration [237] (Fig. 5B). Measuring changes in the venous serum Cl^−^ concentration may provide a simple way to identify the aerobic metabolism status and mitochondrial function, although more direct evidence is needed to conclude this.

Cl^−^ is also responsible for acidification of the lumen of endosomes and lysosomes through the function of the Cl^−^/H^+^ exchange system [238–240]. The acidity of endosomal and lysosomal lumens is involved in a cellular recycling process called autophagy in various cell types [29, 30, 241]. For example, defects in microglial lysosomal acidification lead to impaired autophagy and phagocytosis, causing progressive neurodegeneration and persistent neuroinflammation [241]. Furthermore, autophagy has been reported to decrease with age, and this decrease plays an important role in both the development of age-related diseases and physiological aging [242]. Thus, Cl^−^ plays a key role in autophagy, degeneration, inflammation and aging via acidification of endosomal and lysosomal lumens [242].

## Conclusion

Cl^−^ plays an important role in maintaining electrical neutrality by being transported as counter ions when cations such as Na^+^ and K^+^ are transported. However, little is known about the physiological roles of Cl^−^ other than maintaining electroneutrality. As described in this review article, cytosolic Cl^−^ is an important factor in the regulation of biological functions, possessing various physiological activities. I would like to conclude this review with my sincere hope that the recognition that Cl^−^ itself is an important regulator of various enzymatic activities will spread beyond its significance in maintaining the electroneutrality during cations movements, and that further research on the physiological roles of Cl^−^ will progress.

## Data Availability

The data underlying this article will be obtained via PubMed and Google Scholar.
